# Bridging the Data Gap From *in vitro* Toxicity Testing to Chemical Safety Assessment Through Computational Modeling

**DOI:** 10.3389/fpubh.2018.00261

**Published:** 2018-09-11

**Authors:** Qiang Zhang, Jin Li, Alistair Middleton, Sudin Bhattacharya, Rory B. Conolly

**Affiliations:** ^1^Department of Environmental Health, Rollins School of Public Health, Emory University, Atlanta, GA, United States; ^2^Unilever, Safety and Environmental Assurance Centre, Colworth Science Park, Sharnbrook, United Kingdom; ^3^Biomedical Engineering, Michigan State University, East Lansing, MI, United States; ^4^Integrated Systems Toxicology Division, National Health and Environmental Effects Research Laboratory, United States Environmental Protection Agency, Durham, NC, United States

**Keywords:** *in vitro*, *in vivo*, computational modeling, toxicity pathway, point of departure, extrapolation, risk assessment

## Abstract

Chemical toxicity testing is moving steadily toward a human cell and organoid-based *in vitro* approach for reasons including scientific relevancy, efficiency, cost, and ethical rightfulness. Inferring human health risk from chemical exposure based on *in vitro* testing data is a challenging task, facing various data gaps along the way. This review identifies these gaps and makes a case for the *in silico* approach of computational dose-response and extrapolation modeling to address many of the challenges. Mathematical models that can mechanistically describe chemical toxicokinetics (TK) and toxicodynamics (TD), for both *in vitro* and *in vivo* conditions, are the founding pieces in this regard. Identifying toxicity pathways and *in vitro* point of departure (PoD) associated with adverse health outcomes requires an understanding of the molecular key events in the interacting transcriptome, proteome, and metabolome. Such an understanding will in turn help determine the sets of sensitive biomarkers to be measured *in vitro* and the scope of toxicity pathways to be modeled *in silico*. *In vitro* data reporting both pathway perturbation and chemical biokinetics in the culture medium serve to calibrate the toxicity pathway and virtual tissue models, which can then help predict PoDs in response to chemical dosimetry experienced by cells *in vivo*. Two types of *in vitro* to *in vivo* extrapolation (IVIVE) are needed. (1) For toxic effects involving systemic regulations, such as endocrine disruption, organism-level adverse outcome pathway (AOP) models are needed to extrapolate *in vitro* toxicity pathway perturbation to *in vivo* PoD. (2) Physiologically-based toxicokinetic (PBTK) modeling is needed to extrapolate *in vitro* PoD dose metrics into external doses for expected exposure scenarios. Linked PBTK and TD models can explore the parameter space to recapitulate human population variability in response to chemical insults. While challenges remain for applying these modeling tools to support *in vitro* toxicity testing, they open the door toward population-stratified and personalized risk assessment.

## Introduction

### Next-generation risk assessment

Human health risk assessment of chemical exposures is a complex monitoring, experimental testing, and modeling task, facing uncertainties and challenges at nearly every step of the way along the exposure-to-outcome continuum (1). Its primary purpose of protecting the general public can lead to conservative regulatory recommendations for “safe” exposure dose that may be overprotective in many circumstances. Its practice is based on all related sciences and constantly shaped by emerging disciplines and technological advancements. Advancements in exposure science, biology, toxicology, chemistry, and epidemiology in the new millennium are providing unprecedented opportunities for a multi-disciplinary approach to address a number of the challenges in chemical safety assessment (2). With the increasing use of personal and mobile sensors and deployment of ground and satellite monitors, and along with the rapid technological refinement of analytical chemical equipment, the spatial and temporal resolution of both external and internal exposure monitoring for numerous chemicals have dramatically improved (3, 4). On the epidemiology front, omics-wide measurements of human samples have brought this traditional, health outcome-oriented discipline steps closer to helping reveal the molecular mechanisms of environmentally related diseases (5). In biology, since the completion of the first human genome sequencing, technological revolutions in molecular systems biology have allowed us to peek into the microscopic world of life at genomic, epigenomic, transcriptomic, proteomic, and metabolomic levels simultaneously and make documenting, molecularly, every cell of our body within reach (6). Data streams coming from all these disciplines, at a growing personalized resolution, are converging to revolutionize the way chemical safety assessment is conducted for the betterment of public health.

### *In vitro* assay-based toxicity testing

Among all these ongoing changes, a paradigm shift has been under way since the publication of the influential NRC report, “Toxicity Testing in the 21st Century (TT21C): A Vision and a Strategy” in 2007 (7). This vision advocates the usage of cells or organoids, ideally of human origin, as the primary testing substrates for assessing chemical toxicity and health risk. A number of considerations and forces are behind this dramatic departure from the traditional “gold standard” of using whole animals for toxicity testing. The animal-based approach examining gross apical endpoints has been in place for nearly half a century and its ability to predict human health risk under environmental exposure conditions is very limited with a wide range of uncertainties (8). This adherence to a failing tradition without much progression in decades is in stark contrast to the exponential growth of our knowledge in the basic biological science where the revolutionary advancement of molecular interrogation and manipulation tools is enabling us to examine biological perturbations at unprecedented resolutions and scales. The traditional animal approach does not utilize much our improved understanding of how life works at the level of molecular pathways and networks, which provides the opportunity to gain deeper mechanistic insight into chemical-inflicted health outcomes.

The transformation of toxicity testing is also driven by non-scientific factors. The traditional animal-based testing method is slow and costly, as exemplified by 2-year rodent cancer studies. The low efficiency and high cost generate a huge backlog of untested, existing chemicals, and newly invented chemicals waiting for approval to enter the commercial market (9–11). Last, but not least, there is increasing societal pressure around the ethical use of laboratory animals for human benefit. Multi-decade advocacy for humane treatment of experimental animals through the 3R principles: reduction, refinement, and replacement, has led to the legislative ban of cosmetic ingredient testing on animals in several geographies including EU and India (12, 13). While animal-based apical endpoint studies remain the predominant toxicity testing approach mandated by government regulations, the cell or organoid-based *in vitro* toxicity approach is gaining steady acceptance. The international scientific community has launched a number of initiatives to push for animal alternatives by developing *in vitro* assays (14–16). Inter-agency collaborations such as the Tox21 project develop, standardize and use high-throughput, high-content cell assays to generate highly reproducible screening data (14).

### Limitations of *in vitro* testing approach

While chemical safety assessment based on *in vitro* toxicity testing holds a great promise, with the added benefit of accelerating testing and eliminating animal usage, this approach alone has obvious limitations and faces multiple challenges that have to be overcome to be practically useful (17). Ideally an *in vitro* approach requires the right types of cells or organoids for assays that can closely mimic the physiological environments *in vivo*, including the native extracellular milieu and cell-to-cell interactions normally encountered in a tissue. This is hardly the case as the tissue culturing technology stands today. For chemicals that are bioactivated after passing through the liver or other tissues and thus their metabolites are the actual culprit of toxicity, emulating the bioactivation process experimentally in a quantitatively accurate way can be difficult. With respect to dosimetry, there are challenges in describing the *in vitro* kinetics in the culture medium, where the stability of the test chemicals would affect the cellular responses, and challenges in mimicking the actual chemical kinetics cells in the target tissues experience under real-world exposure scenarios. Defining the proper *in vitro* point-of-departure (PoD) at the cellular level and extrapolating it to *in vivo* apical endpoint alterations, often occurring on a different time scale, is a challenging task. Also nontrivial is the extrapolation of determined *in vitro* PoD chemical dose metrics to *in vivo* exposures. For human population risk assessment, challenges also include compiling an ensemble of cells or organoids to adequately address human individual variability and extrapolating to low-dose effects for environmental exposures. At the 10th anniversary of the 2007 NRC report, a survey conducted among near 1,400 toxicologists and risk assessors revealed that the leading technical barriers to the adoption of the *in vitro* assays-based alternative approaches are, among others, “concerns with regard to the interpretation and extrapolation of the data, failure to capture the integrated whole-animal system, the difficulty in developing dose–response relationships” (18).

Clearly some of the data gaps resulting from these challenges can be partially addressed or overcome through experimental and technological improvement, such as developing organ-on-a-chip and system-on-a-chip technology to better simulate chemical metabolism and physiological interactions, and using cells from a large number of donors to better represent human populations (19–21). However, there are fundamental limitations of the *in vitro* testing framework that cannot be easily improved through experimental means, and would require a computational approach to help bridge the data gaps in a feasible and cost-effective way (22). Indeed, dose-response and extrapolation modeling is a central pillar that was proposed to support the interpretation of *in vitro* assays in the new toxicity testing and risk science paradigm (2, 7, 23). This article aims to provide a glimpse at the computational modeling tools that would help enable *in vitro* assay-based risk assessment, with a primary focus on toxicodynamic modeling of toxicity pathway perturbations.

## Computational approaches for dose-response and extrapolation modeling

### Toxicity pathway and *in vitro* PoD

One of the radical changes after switching to cell or organoid-based testing is that some combinations of *in vitro* measurements will be used as biomarkers to define the PoD instead of the apical, organism-level endpoints normally screened for in animal testing, such as cancer, liver pathology, and reproductive functional disruption. While many traditional biomarker assays, such as those evaluating DNA damage, cellular and organelle states and functions, will continue to be part of the testing repertoire, it is recommended that the selection of *in vitro* endpoints should bear more mechanistic considerations and be organized around the primary toxicity pathways perturbed by the test chemical (7). Toxicity pathways are defined as physiologically functioning biochemical pathways or molecular circuits in the cells that, when sufficiently perturbed by external factors, would lead to adverse health outcomes (7). While the definition is conceptually clear, in practice it is challenging to (a) define the scope of relevant pathways for a particular chemical among the complex intracellular network comprising myriad biochemical nodes and to (b) relate perturbations of the pathways, a subset of the network, to apical endpoint alterations both qualitatively and quantitatively. So far, the culprit pathway(s) known to mediate a chemical's primary toxic effect has been mostly the synthesized knowledge from mechanistic studies. The new toxicity testing approach would require the development of high-coverage assays that screen the entire toxicity pathway space. With the increasing availability and affordability of omics technologies, screening for changes at genomic, epigenomic, transcriptomic, proteomic, and metabolomic levels becomes feasible, which will help identify the most sensitive toxicity pathways for further fit-for-purpose testing (24, 25).

Given a chemical and its initially identified toxicity pathway(s), the next question is what fit-for-purpose *in vitro* assays we need to utilize or develop to cover a sufficient number of biochemical and cellular biomarkers that can truly gauge the degree of pathway perturbation causing an adverse health outcome (25). Cellular perturbation and adversity can be evaluated in a number of traditional as well as novel ways. General cellular health can be monitored by measuring cell viability, LDH release, ROS level, ATP depletion, and mitochondrial membrane potential, etc. Damages of biomacromolecules and cellular components can be assessed by measuring DNA double-strand breaks, DNA adducts, micronuclei, lipid peroxides, and protein adducts etc. Functional assays, such as the beating of cardiomyocytes, metabolic capacity of hepatocytes, and insulin secretion of β-cells, are also available. While these traditional biomarkers provide general, key information about cellular perturbation, toxicity pathway-specific information is also needed. Within the adverse outcome pathway (AOP) framework, toxicity pathways can be measured by examining key events (KE) that occur between the molecular initiating events (MIE) and adverse outcomes (26, 27). KEs emerging immediately downstream of MIEs are sensitive nodes that can be utilized as biomarkers at early time points of perturbations. But as cells adapt through activating downstream KEs, transcriptional, and translational activities can be significantly altered, thus becoming possible candidates for pathway perturbation evaluation. Among all the cellular endpoints—generic cellular status and health, pathway-specific information, specialized cellular functions and activities conducive to the fitness of the organism—choosing the right combinations to sufficiently represent toxicity pathway perturbations that can lead to adverse outcome is a challenge. Further complicating the matter is the determination of the magnitudes and durations of these deviations, which collectively define a cellular PoD that can lead to adverse health outcomes. As discussed in later sections, overcoming these challenges would require mechanistic knowledge of how cells respond and handle chemical stresses at various omic levels of toxicity pathways in the absence and presence of adverse outcome manifestation.

### Computational modeling of toxicity pathways to predict *in vitro* PoD

#### The biasing effect of *in vitro* kinetics on PoD dose metric determination

The majority of cell-based assays are currently done with the test chemical added at a fixed, initial concentration at the beginning of the experiment and then the cells are continuously cultured for a number of hours or days, as exemplified by the ToxCast and Tox21 assays (14, 15). The minimal initial concentration that can drive the tested cellular system to deviate from the basal state for a predefined magnitude and duration deemed to lead to adverse outcomes *in vivo* is often regarded as a PoD concentration for the chemical and toxicity pathway investigated. In the new toxicity testing paradigm, it is assumed that the nominal *in vitro* PoD concentration so obtained is equivalent to the plasma or tissue concentration cells *in vivo* are exposed to that would lead to an adverse outcome (28, 29). This assumption has some caveats. First of all, it ignores the fact that many chemicals are not stable in the culture medium as they can either be metabolized somehow in the medium or move into the cells and are metabolized intracellularly. As a result, the concentration of the chemical in the medium may decay and that in the cells may first rise and then fall over time (30). The biochemical and cellular alterations observed under such experimental protocol are the result of cells responding to a changing concentration of the chemical or its metabolites, which can be very different from the response of cells *in vivo* which can experience fluctuating or relatively constant chemical concentrations depending on the exposure and clearance rate. A second consideration is that many chemicals can bind to either the plastic wall or ingredients in the medium, making the effective free concertation lower than the nominal concertation even when there is no significant clearance otherwise (31). In certain cases evaporation of volatile chemicals may be significant (32, 33). All these considerations above challenge a straightforward usage of initial chemical concentrations in the culture medium as the *in vivo* tissue PoD concentrations. In a situation where a chemical decays rapidly in an *in vitro* assay, the effective or average concentration of the chemical capable of pushing the cells to the observed PoD can be far lower than the nominal concentration. Using the nominal concentration as a PoD dose metric will lead to an underestimate of the health risk of the chemical, a bias that is undesirable and should be avoided by all means. Moreover, in addition to dose, chemical toxicity is also a function of exposure time. In clinical settings, drug toxicity can correlate with accumulative dose such as the case of doxorubicin-induced congestive heart failure (34). Thus combination of nominal concentration and exposure duration has also been used to more comprehensively characterize PoD dose metrics (35). To address the concern of *in vitro* dosimetry selection, Groothius et al. recommended a decision tree—based on the characteristics of the *in vitro* kinetics, cellular endpoint, and mode of action of the test chemical—to help choose among nominal, measured total, free, and intracellular concentrations, time-averaged, and area-under-curve as reported PoD dose metrics (36).

Real-world chemical exposures are complex, leading to dynamical tissue dosimetry. For accidental exposures, the internal chemical concentrations can rise acutely in a short period of time. For chronic environmental exposures, depending on the exposure scenario and clearance characteristics, the internal chemical concentrations can be episodic or relatively constant within a long period of time albeit possibly changing slowly. For clinical applications, drugs are administrated using a variety of regimens, resulting in rapidly fluctuating plasma and tissue concentrations within minutes, hours, and days. Simple *in vitro* assays cannot easily duplicate either of the above scenarios. Microfluidic technology such as organ-on-a-chip devices can have a more controlled delivery of the test chemical to the cultured cells or organoids to better mimic clinical applications and environmental exposures, but currently it is challenging to scale up in a high-throughput, economical manner to cover the chemical space and toxicity pathway space to be tested (37).

#### Constructing and calibrating toxicity pathway models with *in vitro* assay data

**Figure 1:**
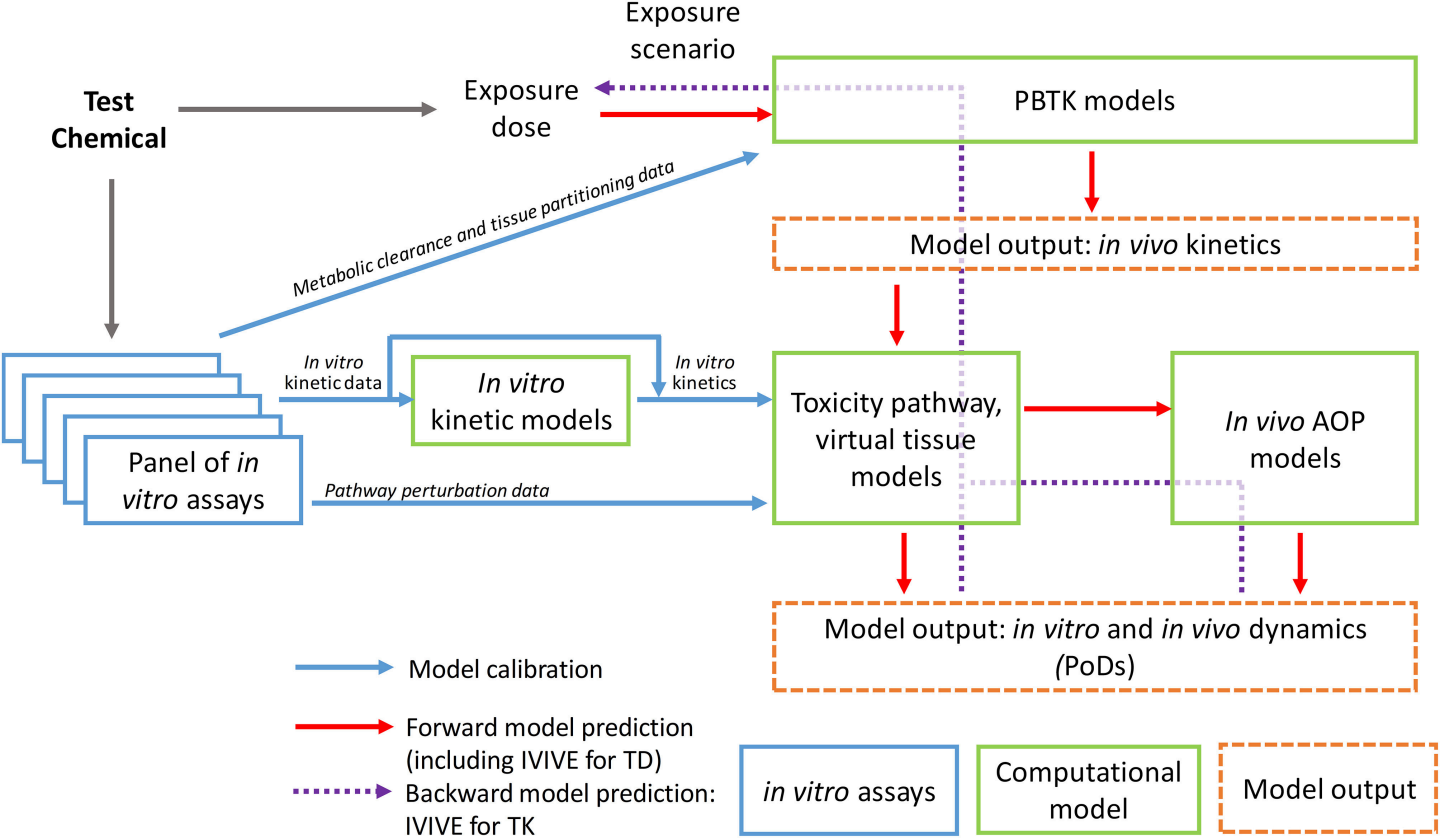
Schematic illustration of the workflow of computational approaches supporting dose-response modeling and *in vivo* extrapolation based on *in vitro* testing data.

To bridge the data gap between *in vitro* and *in vivo* kinetics and correct for the resulting difference in the PoD chemical dose metrics, computational toxicity pathway models and likely *in vitro* kinetic models are needed (Figure 1). The former is to mathematically reconstruct the relevant biochemical circuits operating in the cells as a dynamical system by using coupled differential equations (23). These differential equations mechanistically describe the KEs in the toxicity pathway of the chemical's AOP, including the interactions and regulations between key biochemical nodes across different omic scales. In a dynamical pathway model, model parameters typically include transcription and translation rates, mRNA and protein half-lives, binding affinities for ligand-protein, protein-protein, and protein-DNA interactions, apparent Hill coefficient for ultrasensitive responses, and Michaelis-Menten constants for enzymatic reactions (23, 38–40). A useful pathway model will mechanistically capture the perturbation dynamics of the underlying biochemical circuits from the MIE through downstream signaling events. For *in vitro* testing where organoids or 3-D tissues are used, computational virtual tissue models capturing cell-to-cell interactions that give rise to tissue structures will be needed, especially for understanding developmental processes (41, 42). These virtual tissue models normally use an agent-based modeling approach where the behavior of each agent, representing a cell, can be described by predetermined rules or through mechanistically-based toxicity pathway modeling as described above (43).

To calibrate these toxicity pathway models, the *in vitro* kinetic information of the test chemical is needed as model's input. Therefore the chemical concentration, particularly the free concentrations, in the culture media and cells during the course of the *in vitro* experiment should be determined whenever possible. As high-throughput non-depletive methods of quantifying chemicals in miniaturized assays improve, determining chemical concentration kinetics is becoming increasingly practical (44). In some cases, an *in vitro* kinetic model describing the chemical concentration changes in the culture medium and cells over time can be constructed (Figure 1). Research efforts have been under way to construct *in vitro* kinetic models for validating and predicting chemical concentration variations in widely used cell lines (30, 45–47). The Virtual Cell Based Assay (VCBA) project in Joint Research Centre in European Union aims to predict time-dependent concentrations of a test chemical both in the culture medium and within the cells by using ordinary differential equations that incorporate the physicochemical properties of the chemical and metabolic and cellular properties of the cell lines. The determined free concentrations in the culture medium can be different from the nominal concentrations by several orders of magnitude, emphasizing the importance of characterizing chemical distribution in *in vitro* assays (46, 47). These *in vitro* kinetic models can be used to predict cultural and intracellular chemical concentration changes over time for initial chemical dose or repeated dose not experimentally tested. The *in vitro* kinetics predicted or actually measured will then be used as input to calibrate the toxicity pathway or virtual tissue models. The model output will be the dynamics of the key biochemical nodes and other cellular metrics identified collectively as the PoD biomarker set, many of which are experimentally measured with the *in vitro* assays. Model parameters are calibrated through minimizing the difference between the model output and *in vitro* assay data obtained at different time points for different initial concentrations of the chemical tested. This calibration is essentially a training process for the dynamical toxicity pathway model.

An ultimate goal of constructing the *in vitro* kinetic and toxicity pathway models and calibrating them based on data from the *in vitro* assay protocols is to help identify the true *in vivo* PoD chemical concentrations or other relevant dose metrics in target tissues under realistic or anticipated human exposure scenarios (Figure 1). For certain environmental exposure settings where the exposure is chronic and the chemical clearance is slow so its plasma and tissue concentrations are relatively constant, static chemical concentrations can be virtually applied to drive the calibrated computational toxicity pathway models to reach or surpass the predetermined PoD to predict the *in vivo* chemical concentration in the target tissue that would lead to an adverse outcome. For clinical applications or other environmental exposure settings where the chemical tissue concentrations are episodic and constantly changing, dynamic chemical concentrations mimicking these conditions, likely aided by toxicokinetic models, will be applied to the computational toxicity pathway models to predict the dosing scenario that would result in the predetermined toxicity PoD in the model (48).

### Determining and modeling molecular KEs of toxicity pathways and PoD

To determine appropriate *in vitro* PoDs in the new toxicity testing framework, identifying key, experimentally measureable biochemical and cellular events is a critical step. The choices of *in vitro* PoD have been so far arbitrary and many of which are chosen for convenience due to conventional usage. For example, AC_50_, and IC_50_ are routinely used to evaluate chemical effects on signaling events such as receptor binding, reporter gene expression, enzyme inhibition, as well as ATP depletion and cell viability. Many follow-up studies analyzing the ToxCast and Tox21 data focused on the concentration point giving rise to 50% of the maximum response (49–52). While using these biomarkers are valuable in ranking the potency of chemicals and prioritizing them for further testing, their usage as *in vitro* PoD to infer adverse health outcome is questionable due to lack of quantitative correlations. In certain cases, the benchmark-dose (BMD) approach or its variants are used to define *in vitro* PoD (53). This approach considers the shape of the entire *in vitro* concentration-response curve and variability. While an improvement over using AC_50_ and IC_50_, the choice of BMD levels, such as BMD_10_, BMD_20_, can be arbitrary and face a similar issue of relating to *in vivo* effects.

Moreover, changes in many of the cellular PoD metrics are not only a function of chemical concentrations, but also a function of the duration of chemical treatment. An obvious example is the cell viability assay, where incubation with a chemical for a longer time generally results in more cell deaths (35). This time-dependency generally leads to a left-ward shifting of the concentration-viability curve, and as a result, a different PoD concentration regardless of using IC_50_ or BMD_10_. For many transcriptionally-mediated cellular stress responses, induction of the stress genes peaks at various times depending on the genes and chemical concentration, thus concentration-response data obtained at a single time point can barely represent the overall response profile (39, 54). Therefore, it is unrealistic to define a PoD solely based on a single cellular metric obtained at a single time point. Rather, PoDs should contain dynamic information, i.e., both the magnitude and duration of the changes of the relevant biomarkers that can adequately represent the sufficiently perturbed cellular state that would ultimately lead to adverse outcomes *in vivo*.

#### Modeling adaptive cellular stress response for PoD

Defining the scope and degree of cellular perturbations that culminate in adverse outcomes is a challenging task requiring basic knowledge on biological robustness and homeostasis, such as how cells handle stress to continuously function and survive in the face of adversity (55). Adaptation is a salient feature of biological systems and is often framed as a determinant of PoD when the adaptive capacity is reached (7). It is commonly thought that if cells have adapted to the stress posed by a chemical and as a result restored cellular homeostasis, the chemical at the tested concentration can be considered safe. This view however poses many questions about the relevance of using a PoD so derived in human risk assessments. The immediate question is what aspect of the cellular state should be looked at to determine whether and how well the cells have adapted. Is it the general cellular heath state, stress pathway status, cell growth or the cell's specialized function? If cells have adapted well with the general cellular heath state (as measured by biomarkers such as ROS, ATP, viability, and LDH) largely indistinguishable from unstressed cells, are their specialized functions equally preserved or nonetheless compromised (56)? Shah et al. has used multiple cellular biomarker data deposited in the ToxCast database, including those representing general cellular health and activation of specific stress pathways, to represent cellular state trajectories in response to chemical perturbations (57). In the study, cells with trajectories that move irreversibly away from the basal state were deemed to exceed a tipping point while those with recovering trajectories are still within or returning to the bound of the normal state. It is unclear though whether these “recovering” cells have or will completely reset their states from the chemical stress or would retain some long-term memory that would affect their response to future insults. It has been shown that epigenetic changes can occur after recovering from stress, which can be inherited through several cell generations to transcriptionally affect future stress response, as demonstrated in *C. elegans* that undergo heat stress (58). It is unclear whether this post-stress epigenetic memory is a general phenomenon.

**Figure 2:**
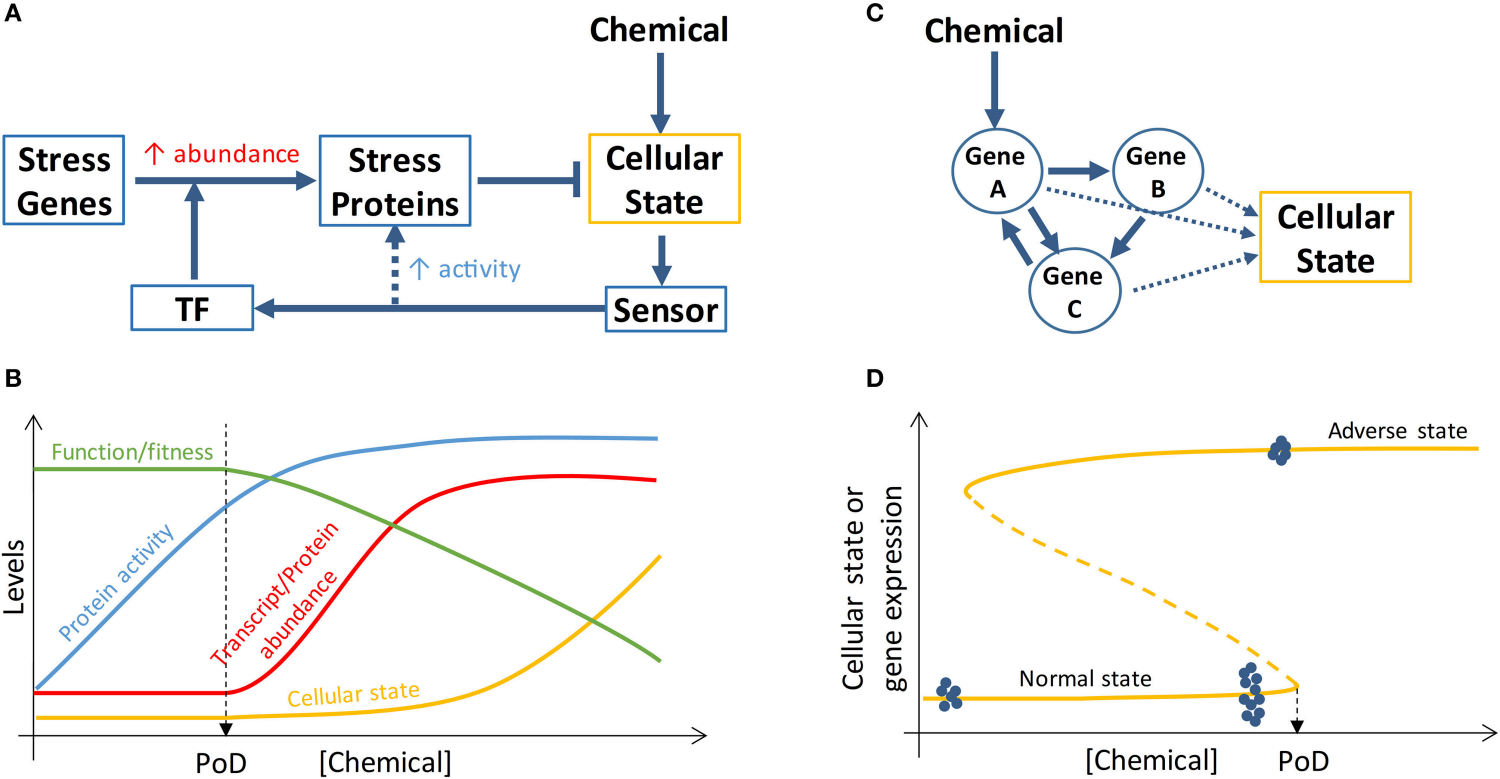
Schematic illustration of PoD resulting from perturbation of stress pathways and bistable gene networks. **(A)** A simplified view of a cellular stress response network where posttranslational control (dashed arrow) increases the activities of stress proteins and transcriptional control activated by transcription factor (TF) increases the abundance of stress proteins. **(B)** Chemical concentration-dependent transition of the stress response. At low chemical concentrations, the activities of basal, preexisting stress proteins are augmented (through posttranslational control) to maintain homeostasis of the cellular state and specialized cell function and fitness are intact. When the chemical concentration reaches a level that maximizes the activities of the preexisting stress proteins, transcriptional control is initiated to increase the abundance of stress proteins to continue to maintain homeostasis and survival. The onset of transcriptional control may define a PoD because specialized function/fitness may be compromised due to translational and metabolic reprogramming (not shown and refer to main text for details) associated with transcriptional control. **(C)** Cellular phenotype can be determined by the state of a gene network which can be perturbed by chemicals. **(D)** If the gene network forms a bistable switch, the bifurcation point of the network defines a PoD. For chemical concentrations below the PoD concentration, cells remain in the normal state; for chemical concentrations above the PoD concentration, cells switch to the adverse state. Gene expression levels can exhibit different variabilities depending on the cellular state. When normal-state cells are exposed to chemical concentrations well below the PoD concentration, the variability of gene expression within the bistable gene network is small (blue dots on the far left of the curve, representing gene expression levels in single cells). When normal-state cells are close to the PoD, gene expression variability dramatically increases (blue dots close to PoD). Once cells switch to the adverse state, gene expression variability decreases again (blue dots on the top curve). Refer to main text for further details.

Transcriptional induction of suites of stress genes is the hallmark of cellular stress responses to chemical insults and has been proposed as a general method for chemical surveillance and *in vitro* screening (59). A typical stress pathway (Figure 2A) involves a sensor molecule to detect the cellular state change, a transcription factor that can be specifically activated by the senor molecule or by the state change directly, and a battery of stress proteins which are induced transcriptionally and participate in reactions that restore the perturbed cellular state, such as altered ROS, DNA damage, ATP levels, to homeostasis (59). These canonical stress response pathways are organized primarily as negative feedback loops and have been constructed into mathematical pathway models to understand their dose-response behaviors (60–63). With an integral control or proportional control with high loop gains, the cellular state maintained by the feedback loops can exhibit threshold phenomena in response to chemical perturbations, where the threshold point corresponds to the stress intensity that causes the maxing out of the induction of stress genes (60, 64). This threshold chemical concentration where maximal induction of stress genes occurs often demarcates the beginning of deterioration of general cell heath as determined by assays such as cell viability and LDH release, suggesting that stress gene expression that increases concurrently with increasing chemical stress intensity is responsible for maintaining cell survival (39, 54). If cell survival is used as a biomarker for PoD, a mathematical model of the relevant stress pathway can be constructed and calibrated to predict PoD by simulating maximal stress gene induction.

Despite the above considerations, it is questionable whether loss of adaptation as a result of gene induction saturation and subsequent decline in cell survival can be used as proper biomarkers for predicting *in vivo* adverse outcomes (65). Emerging evidence suggests that they may be too insensitive for this purpose for reasons below. Cellular stress response is a highly energy-demanding process, as a number of stress proteins are being synthesized *de nova* to levels several to tens of folds of their basal levels (66–71). Bioenergetically constrained, cells under stress initiate global translational reprogramming and metabolic reprogramming to preserve energy for the stress response for cell survival while sacrificing other non-survival-essential programs. In sufficiently stressed cells, the translation of most proteins is inhibited or shutdown through stress-induced posttranslational modification of translation initiation factors such that the normal 5′ cap-dependent translation initiation becomes compromised (72–74). In contrast, the mRNAs of many essential stress proteins (such as antioxidant enzymes, DNA repair enzymes, heat shock proteins in the canonical stress pathways) are continuously being translated because they contain alternative internal ribosome entry sites (IRES) in their 5′ untranslated regions, permitting ribosome assembly and initiation of cap-independent translation at these IRES sites (75). Through this mechanism and others, the induction of stress genes is unaffected (76). However, due to the global translation inhibition, the synthesis of proteins involved in specialized cell functions (which are unlikely essential to cell survival at the moment of stress) is likely halted. In addition to translational reprogramming, metabolic reprogramming may also occur to optimize energy distribution in the face of cellular stress (77, 78). In the case of oxidative stress, the flux through the pentose phosphate pathway is promoted to synthesize more NADPH as reducing agent to handle the stress, which takes the carbon flux away from the glycolysis and downstream Krebs cycle, resulting in less energy available for other cellular processes (79). Therefore, as a result of stress-induced translational and metabolic reprogramming that accompanies the onset of transcriptional induction of stress genes, cells enter a survival mode to avoid being killed, with their specialized functions and other nonessential activities sacrificed or suspended temporarily (80). This mode of cellular stress response suggests that the beginning of transcriptional induction of stress genes may define a fitness-relevant PoD (Figure 2B). In keeping with this notion, a recent ecological study with brook trout, a cold-water fish, showed that the threshold water temperature inducing the onset of heat shock protein expression in the gill is consistent with the average water temperature limiting the fish population in the field, suggesting a connection between thermal stress response and fitness (71).

This mode of action raises the question on the mechanisms cells utilize to counter stress in the absence of transcriptional induction of stress genes. When exposed to mild and/or transient stresses, cells do not necessarily and sometimes are unable to launch transcriptional responses (81–83). For one, this is because stress response through transcription is too slow to be initiated to handle transient, but sometimes detrimental, stresses (65). Moreover, the transcriptional network can be insensitive to low-level stresses due to the relatively small feedback loop gain near the basal condition where transcription factor-independent constitutive expression of stress genes can be dominant (60). Instead, there appear to be a number of posttranslational control mechanisms cells can engage to promote their anti-stress capacity, by activating pre-existing stress proteins that are normally inactive (Figure 2A) (65). The activation can be both through allosteric regulation or posttranslational modification such as phosphorylation, oxidation, and acetylation (84–86). These processes are fast in responding and demand much less energy than *de novo* protein synthesis. Therefore, it is possible that cells adapt to stresses through a two-tiered process depending on stress intensity (65). At low stress levels, cells use posttranslational regulation of basal, pre-existing stress proteins to enhance their activities to maintain homeostasis while their specialized functions are unaffected because there is no global translation inhibition and metabolic reprogramming yet (Figure 2B). At higher stress levels, all the pre-existing stress proteins are activated and their abundance needs to be increased—through initiating transcriptional induction—to continue to maintain homeostasis and survival. Concomitant with the initiation of transcriptional control, cells enter survival mode, and stress-induced translational and metabolic reprogramming diverts energy and molecular resources away from the maintenance and synthesis of non-survival-essential proteins, including those executing specialized cellular functions (56). At very high stress levels where transcriptional control is maxed out, cell viability starts to decrease with increasing cell death.

Therefore, the transition or switching of cells from posttranslational control to transcriptional control may demarcate a functional PoD, where specialized cell functions start to deteriorate or other activities conducive to fitness starts to slow or pause. As a result of this two-tiered operation, it may require us to shift the monitoring focus from measuring transcriptional changes to measuring posttranslational modifications as biomarkers to more sensitively detect PoD that is associated with functional alterations. Concomitantly, computational toxicity pathway models describing both the short, fast posttranslational feedback loop and the slow, transcriptional feedback loop need to be constructed to simulate the transition from posttranslational to transcriptional control. Such pathway models, calibrated based on *in vitro* assays measuring transcriptomic and proteomic responses, will allow us to extrapolate functional PoD for tissue chemical concentrations encountered in real world exposures.

#### Transcriptomic alterations and apical adverse outcomes

The idea that transcriptional alteration can be associated with adverse outcomes has also emerged recently from toxicogenomic studies. Since the RNA microarray technology and subsequently next-generation sequencing became available and affordable, many animal studies have been conducted to examine transcriptomic changes at confirmed or suspected target organs or tissues in response to chemical exposures (87–89). Many of the tested chemicals are legacy chemicals, such as pharmaceutical compounds and well-studied environmental toxicants, with known apical endpoint toxicities. The primary purpose of these toxicogenomic studies was to explore whether detecting transcriptomic changes within a short exposure time and with fewer animals can replace the traditional apical endpoint tests. Initially it was thought that transcriptomic changes may be a very sensitive biomarker for this purpose, i.e., they may occur at earlier time points and at lower doses than required for apical endpoints to manifest. However, the vast majority of these studies have shown, surprisingly, that there is basically a temporal and dose concordance between transcriptional changes in the most sensitive pathways and the adverse health outcomes in the animals, for both cancer and non-cancer endpoints (90–95).

These findings are in agreement with the two-tiered cellular stress response profile discussed above, where if a chemical dose is high enough to induce transcriptional alterations, the cell's specialized function or other activities conducive to the fitness of the organism may be compromised, resulting in adverse outcomes. While toxicity pathway alteration at the transcriptional level can be both the cause and result of adverse outcomes, these toxicogenomic studies suggest that examining transcriptomic changes may not be early and sensitive enough to predict and avert toxicity. Although these studies were done primarily in animals, they provide important clues to guide toxicity pathway-based *in vitro* testing, with respect to the omic tiers that should be examined. For chemicals inducing cellular stress responses, proteomic and metabolomic changes may act as sensitive biomarkers for detection.

#### Modeling critical state transition through bistable gene networks

Transitioning of toxicity pathways and AOPs from normal to adverse states, as driven by an MIE such as receptor binding, can be regarded in some cases as switching of the underlying dynamical system from one attractor state to another (23). Converging evidence from many fields, including physics, ecology, climate, finance, biology, and psychology, has revealed that certain generic features exist in complex dynamical systems and can be exploited to predict the imminence of abrupt state transitions such as species vanishing in ecosystems, financial crisis, or climate change (96, 97). Although often driven by slowly-varying external or internal factors, the transition can occur abruptly through a saddle-node or other types of bifurcation corresponding to a tipping point (98). A common feature of a dynamical system that is approaching such tipping point is that its rate of recovery from small, transient perturbation by random noise slows down dramatically (99). As a result of this critical slowing down, the state of the system (i) displays an increased temporal auto-correlation and (ii) fluctuates more dramatically (increased variation) around the current attractor state. Both of these features have been utilized to successfully predict critical transition in many fields (100–103). Stable cellular fates or phenotypes can be regarded as attractor states of a complex dynamical system underpinned by gene regulatory networks (Figure 2C) and transitions between cell states are driven by physiological signals or exogenous chemicals (104). It has been postulated that cancers are the result of normal cells falling irreversibly into cancer attractor states that are either preexisting or created by mutations or other carcinogenic drivers (105). As chemical concentration increases or their immediate effects (such as mutations) accumulate in cells/tissues, driving the gene network close to the tipping point, based on the theory of critical slowing down, the gene transcripts and proteins, which are constantly perturbed by stochastic gene expression noise (106), would be sluggish to return to their deterministic steady-state levels and thus become more variable (Figure 2D). As a result, the expression pattern of these network genes will exhibit certain statistical features near the tipping point (102, 103, 107), despite that their mean expression levels can remain largely indistinguishable from the normal, healthy attractor state: it is expected that (i) gene expression variance will increase dramatically across time and between cells; (ii) expression correlation between genes in the same network will increase dramatically due to mutual feedback regulation; (iii) similarity between individual cells, defined by the gene expression vector, will decrease. So the pre-tipping point can be predicted by a composite index derived from the above statistics of network genes. Practically this can be achieved by analyzing gene expression data obtained from *in vitro* RNAseq or real-time RT-PCR assays performed at single-cell resolution.

Critical transition of a biological system from a healthy attractor state to a diseased attractor state driven by environmental exposures can be irreversible such that even the external and/or internal exposure recedes to a lower level, the affected system is still locked in the diseased state (Figure 2D). In dynamical systems theory, such irreversibility may be mediated through a bistable switch where the system can adopt one of the two possible states under the same conditions (98). The primary network motif of bistable switches is a positive or double-negative feedback loop. Such feedback loops in gene networks have been shown to play essential roles in mediating many cellular phenomenon, including proliferation, differentiation, and apoptosis (108). It defines an unambiguous threshold beyond which the cellular system will switch irreversibly to a different state, which can be another physiological state, or in the event of toxicity, an adverse outcome state. Environmental and pharmaceutical chemicals have been shown to interfere with these bistable-switching processes (38, 109). Computational modeling of these feedback networks capable of alternative, contrasting attractor states is important for interpreting single-cell resolution transcriptomic and proteomic data and predicting PoD for chemicals perturbing bistable-switching toxicity pathways.

### *In vitro* to *in vivo* extrapolation (IVIVE) modeling

For human risk assessment based on the advocated *in vitro* testing approach, an obvious challenge is how to interpret these *in vitro* assay results in the *in vivo* context and inform safety assessment for human populations. This task would rely on computational IVIVE modeling for both toxicodynamics and toxicokinetics.

#### IVIVE modeling for toxicodynamics of AOPs

The biological scope of an AOP can vary depending on the chemicals and their adverse outcomes. Some AOPs can be very local, i.e., the MIEs and the apical endpoints occur within the same target organs or tissues without much involvement of other parts of the body. For these localized disease outcomes, for instance, cancer and certain liver toxicity, the *in vitro* PoD dose metrics derived from cell or organoid-based assays may be directly applicable to *in vivo* situations with minor adjustment. For disease outcomes clearly involving whole-body physiology, such as disorders in blood pressure, body temperature, hormones, metabolism, and immunity, perturbations at one target site can be systemically compensated through feedback or feedforward regulations that involve other body parts to maintain homeostasis. For example, the local effect of an endocrine disrupting chemical that inhibits thyroid hormone secretion can be compensated, within a limit, by the hypothalamic-pituitary-thyroid (HPT) axis feedback through enhancing TSH secretion (110). It is conceivable that an isolated, *in vitro* assay using thyroid follicles to measure inhibition of thyroid hormone synthesis/secretion will not be able to inform—by itself—the degree of deviation in hormone levels *in vivo*, including the expected increase in TSH, which can have a proliferative effect on the thyroid gland. While this PoD gap between *in vitro* and *in vivo* situations may be addressed to some extent by future organ-on-a-chip technology that can create a mini HPT system, dynamic computational models of the endocrine feedback axis will be able to bridge the gap by taking the *in vitro* assay results as the input and predict *in vivo* hormonal changes as the output. Computational HPT models for both rats and humans have been constructed and applied to predict the *in vivo* effects on thyroid hormones of several thyroid disruptors such as thyroperoxidase inhibitors and sodium-iodide symporter inhibitors (111–113). A computational model of the endocrine system can also simulate cyclic hormone dynamics and incorporate population variability into the relevant physiological processes to conduct risk assessment based on *in vitro* testing data. Bois et al. has recently linked ToxCast aromatase inhibition data to a menstrual cycle model to predict cycle length changes in women for chemical mixtures (114). Therefore, for the risk assessment of endocrine disruptors, we should aim to model endocrine systems by integrating different biological scales of the relevant AOPs and utilize these models to predict *in vivo* effects by taking *in vitro* toxicity pathway perturbation data (115). Developing these quantitative AOP models capable of risk prediction will aid regulatory decisions (116). Similarly, in the pharmaceutical research front, quantitative systems toxicology plays an emerging but important role in predicting drug toxicity to support drug development based on *in vitro*, animal, and clinical studies. As the R&D cost continues to escalate exponentially, the need for reliable IVIVE in toxicodynamics and pharmacodynamics using cell culture data at the early stage of drug development ever increases (117). To this end, many mathematical models describing the underlying pathophysiology of drug adverse effects have been developed for predicting renal toxicity, gastrointestinal toxicity, arrhythmia, and liver injury (118–120).

#### IVIVE modeling for toxicokinetics

Compared to the nascent effort in toxicity pathway and systems toxicology modeling, toxicokinetic or pharmacokinetic modeling of chemical fates within the human or animal bodies has been around for a number of decades. Physiologically-based toxicokinetic (PBTK) or pharmacokinetic (PBPK) modeling has been used to understand and predict the absorption, distribution, metabolism, and excretion (ADME) for environmental and industrial chemicals since the 1970s (121). Challenges abound, PBTK models play an increasingly important role in chemical health risk assessment (122). More recently, PBPK modeling has seen a resurgence in the pharmaceutical industry, as drug developers aiming to achieve more accurate efficacy and safety are increasingly focusing on compound concentrations in the target tissues and cells and in the meantime hoping to achieve individualized precisions (123, 124). Neither tissue-specific chemical concentrations nor inter-individual variability can be easily addressed with traditional, compartmental PK models; in contrast PBPK or PBTK models are structurally superior by modeling chemical ADME based on human anatomy and physiology. PBPK and PBTK modeling has been traditionally used in a forward fashion, i.e., given a chemical exposure, these models can predict the tissue concentration dynamics of the parent chemical and its metabolites. Since the publication of the 2007 NRC report advocating for *in vitro* assay-based toxicity testing, applying PBTK models to reverse dosimetry, i.e., IVIVE of toxicokinetics, has become an active research area (125, 126). Researchers are eager to apply PoD concentrations derived from low- or high-throughput *in vitro* assays to existing or newly developed PBPK models to back-extrapolate external exposure levels that would produce equivalent target tissue concentrations (Figure 1). As an essential computational modeling component in the TT21C framework, toxicokinetic IVIVE is finding its applications in both environmental chemical risk assessment and drug development based on results from *in vitro* assays (29, 117). Due to space limitation, we would not dwell on the technical details of toxicokinetic IVIVE through PBTK modeling. Readers can refer to excellent reviews indicated above.

### Population variability modeling

In addition to labeling chemicals by their potential hazards, the ultimate goal of toxicity testing is to provide safety assessment for the human population, protect the majority of the public by regulating exposure limit of chemicals of concern, and provide tools for personalized risk assessment. Variations in human individual susceptibility to environmental challenges can be due to both intrinsic and extrinsic factors, including differences in genetics/epigenetics, preexisting disease conditions, nutrition, life stage, co-exposed chemicals, and exposomic history (127). Thus, addressing the issue of human population variability in response to environmental exposures is a complex integrating process involving convergence of multiple data streams in the above areas (128). If relying on *in vitro* human cell and organoid studies mainly, how can we inform public health risk better than applying the default 10 or 100-fold uncertainty factors that are routinely used for inter-species extrapolation from animal studies?

#### *In vitro* approach for individual variability

The data gap and challenge in using *in vitro* assays to inform population health risk are multi-faceted. First and at least for now, most of the *in vitro* assays in development utilize existing cell lines of either animal or human origin. Many of these cell lines are mutant, cancerous cells that have been immortalized or otherwise transformed, so it is questionable how representative they are for an average response of healthy individuals. Despite this caveat, efforts have been under way to utilize human Epstein-Barr virus (EBV)-transformed lymphoblastoid cell lines derived from individuals in the 1,000 Genomes Project. These diversity cell lines are routinely used in the pharmaceutical industry for drug screening (129), but a subset of them were tapped, through using high-throughput assays measuring cytotoxicity, ATP, and caspase levels, for understanding human population variability in chemical toxicity (130, 131). More recently, the effort has been further expanded to include over a thousand cell lines representing nine populations from five continents to characterize human inter-individual variability for a number of chemicals (132). These studies, while not only characterizing the response variability, also help to identify through genome-wide association analysis the primary gene polymorphisms responsible for the heterogeneous cellular responses.

Compared to immortalized human cell lines, primary human cells are closer to mimicking *in vivo* conditions. Numerous studies have been conducted demonstrating individual variability using primary cells. For instance, umbilical cord blood-derived cells were exploited to examine individual variability in the proliferative response to low-dose radiations (133, 134). Primary human B lymphocytes from different donors were utilized to characterize the inter-individual variability in the immunotoxic effect of dioxin on the antibody secretion response and to ascertain how the variability may affect the linearization of the population-average dose response curves (21). Many of the studies with primary human cells use blood cells for their easy access; lack of easy access to solid living human tissues limits the usage of primary cells for toxicity testing. In the past decade, however, with the discovery of induced pluripotent stem cells (iPSC) and maturing experimental protocols to differentiate iPSCs to desired cell lineages, the opportunity of addressing human individual variability and susceptibility using *in vitro* approaches has improved considerably (135). For instance, recently iPSC-derived cardiomyocytes from healthy donors have been used to study the inter-individual variability in the cardiotoxicity of pharmaceutical compounds (136). While iPSC-derived cells may not be able to fully recapitulate the behavior of the corresponding cells *in vivo*, as our understanding of the cell differentiation process improves and the differentiation protocols are further refined, this approach presents the most promising future in generating—in large quantity—different types of cells representing different human races and individuals.

#### Modeling individual variability in toxicodynamics

Regardless the availability of cells representing human populations, to address population variability experimentally, the testing space as defined by the product of individuals, cell types, toxicity pathways, key events, and chemicals is astronomically huge and can be economically prohibitive. Developing experimental assays and further reducing their costs aside, it is appealing and more economical to use computational modeling of toxicity pathways, virtual tissues, and AOPs to explore individual variability and human population risk. In theory, once an average, mechanistically-based model for a toxicodynamic process is developed and validated, individual responses can be cheaply explored computationally by varying relevant model parameters based on defined distributions. Each combination of parameter values randomly drawn from these distributions would represent a human individual. The challenge lies in how to come up with the parameter distributions that can represent a human population. Depending on the granularity of the computational model, i.e., how much biochemical details it describes, model parameters can either have real physical representations, such as binding affinity between a ligand and a receptor, or describe nominal ones as a composite for a number of processes or factors not explicitly modeled. For a toxicity pathway model simulating cellular responses, parameter differences between cells from different individuals can be due to genetic, epigenetic differences which affect the transcription, translation, degradation, and activity of the proteins involved. For organism-level models, such as the endocrine system, individual difference can be captured in parameters governing hormone synthesis, release, metabolism, and feedback regulation, etc. (137). To determine the most important parameters contributing to individual variability, sensitivity analysis can be performed to scan for parameters that affect the model behavior the most (138). In addition, key determinants for heterogeneous individual responses can be gleaned from genome-wide association studies (GWAS) and epigenome-wide association studies (EWAS) of *in vitro* assays that identify differences among individuals in key genes and the modifications in their regulatory regions (132, 139–141). Such information can be incorporated into relevant computational models to explore population variability *in silico*. GWAS and EWAS studies can also help identify important information on joint distributions of certain physiological parameters, which occur through co-evolution or influence by some common yet unknown factors.

#### Modeling individual variability in toxicokinetics

Compared to toxicity pathway modeling of toxicodynamics, simulating toxicokinetics through PBTK modeling to recapitulate population variabilities is in a far more advanced stage. This is largely because (1) individual variabilities in anatomical parameters, such as body weight, cardiac output and tissue volume, which play a significant role in the ADME of a chemical, can be well documented and defined in a human population. (2) A PBTK model generally has far fewer biochemical parameters besides those concerning enzyme and transporter kinetics, which can be experimentally determined more easily. Commercial PBPK/TK simulation software such as Simcyp often has a built-in capability to simulate a virtual population with pre-stored physiological parameter distributions describing body weight, organ weigh and volume, cardiac output and tissue blood flow for difference life stages and races (142–144). Recently population variabilities in the TK of a number chemicals have been explored by using physiological parameters and biomonitoring data from the National Health and Nutrition Examination Survey (NHANES) (145). As far as chemical-specific parameters are concerned, their distributions can be estimated in several ways (127, 146). For human data-rich compounds, such as drugs, these parameter distributions can be back-calculated in a posterior fashion. For parameter-rich compounds obtained through experimental measurement, distributions can be assumed *a priori*. Lastly, with a Bayesian PBTK modeling approach, prior parameter distributions can be further constrained through comparing PBTK model output with the measured chemical tissue concentrations to arrive at posterior distributions that are less uncertain (147, 148).

#### Linking toxicokinetic and toxicodynamic models

The full power of computational modeling lies in combining TK and TD models to address the exposure-to-outcome continuum along the aggregate exposure pathway (AEP) and AOP frameworks (149, 150). A population PBTK/TD model will draw parameter values from defined distributions to simulate the responses of virtual human individuals to dynamic chemical inputs gathered or predicted from exposure studies (151). To this end, epidemiological data such as those from the NHANES database and cohort studies can be utilized to provide information on the internal exposure and endogenous biomarker levels in the same individuals for constructing and calibrating population PBTK/TD models (111). Recently Bois et al. has demonstrated that linking exposure model, TK model, and dynamic hypothalamic-pituitary-ovarian model can be used to predict disruption of the menstrual cycle by aromatase inhibitors in women populations (114). Combined mechanistically-based population PBTK/TD model can also facilitate estimating mixer effects (152, 153). For instance, the PBTK submodel can simulate the chemical-chemical interaction effects due to cross-induction of metabolism. The TD submodel can simulate toxicological responses to mixers due to cross-talk between different toxicity pathways or different MIEs converging on the same pathways.

## Challenges in computational modeling

While mechanistic computational modeling holds great promises to bridge the data gaps from *in vitro* testing to chemical safety assessment, this approach itself faces its own challenges. Uncertainty in parameters and model structures is one of the primary obstacles to constructing a reliable model. Ironically, given all the high-throughput and high-content omics technologies that catapult biological research into the big-data era, efforts to characterize biochemical parameters are still sparse and sporadic. There are no concerted, systematic efforts in the research community to map out biological parameters so far. While a lessor issue for PBTK modeling as the structure of a PBTK model is relatively fixed and it involves far fewer parameters with many physiological parameters well defined, the lack of parametric information has limited the growth of toxicity pathway models into large-scale network models that can better represent human physiology. Technological advancement has allowed global measurements of protein abundance, synthesis rates, and half-lives in the cells, but more needs to be done to enable systems biology pathway modeling (154, 155).

The majority of mathematical models at biochemical pathway levels are constructed to simulate the behavior of an averaged cell population, as often reported in bulk cell assays measuring aggregated responses. Yet, individual-cell behaviors can be far different than the average, especially for switching responses where cells often diverge into two distinct subpopulations while the cell population average is more graded (156). Cell-to-cell variability due to stochastic gene expression can obscure the deterministic threshold theoretically predicted by computational toxicity pathway models. Therefore, under this situation the effect of cell-to-cell variability in toxicity pathway modeling needs to be account for, before individual human variability is considered. Moreover, for a bistable gene network, the switching behavior can be random due to gene expression noise, a phenomenon that a deterministic model using ordinary differential equations cannot easily reproduce (40). Thus, to identify the PoD of bistable gene networks, by utilizing the gene expression variance and correlation metrics as discussed above, a stochastic modeling approach simulating individual cells will be more helpful. As single-cell transcriptomic analysis becomes feasible, data are available for calibrating these stochastic single-cell models. However, stochastic modeling algorithms are computational intensive as they simulate reactions one step at a time (157). This would challenge the IT infrastructure for achieving simulations in realistic time frame. Moreover, simulations at single-cell resolution are also needed in virtual tissue modeling to help interpret organoid-based assay results. Biosimulations at this scale will need to account for not only single-cell behavior, but also interactions between different cells. Therefore, as biochemical pathway modeling becomes more sophisticated, balancing between sufficient computational description of the underlying biology and realistic simulation time can be a challenge.

## Concluding remarks

As the financial and intellectual investment in *in vitro* toxicity testing continues to increase worldwide, it is time to move beyond priority screening and develop, on top of omic-wide studies, fit-for-purpose assays. These toxicity pathway-focused assays need to be complemented by computational modeling to extrapolate the *in vitro* findings to real-world, *in vivo* dose-response outcomes. Mechanistically-based mathematical models of *in vitro* and *in vivo* kinetics, toxicity pathway perturbations, virtual tissue dynamics, and *in vivo* AOPs constitute the founding pieces for this aspect of next-generation risk assessment (158–160). Despite many experimental and computational challenges remaining, the usage of these models, either alone and in combination, will not only help make more reliable predictions for *in vitro* and *in vivo* PoDs, but also for the heterogeneous human population responses. As the genetic and epigenetic information of individuals becomes available and is incorporated into these mechanistic models, they open the door toward population-stratified and personalized risk assessment.

## Author contributions

QZ wrote the initial draft of the manuscript. JL, AM, SB, and RC all contributed to the ideas discussed in the manuscript and edited the manuscript critically.

### Conflict of interest statement

JL and AM are employed by Unilever. The remaining authors declare that the research was conducted in the absence of any commercial or financial relationships that could be construed as a potential conflict of interest. Mention of products and trade names does not indicate endorsement by the federal government. Conclusions drawn in this study neither constitute nor necessarily reflect U.S. EPA policy.
